# Rapid Identification of Pseudomonas aeruginosa International High-Risk Clones Based on High-Resolution Melting Analysis

**DOI:** 10.1128/spectrum.03571-22

**Published:** 2023-01-11

**Authors:** Kristyna Dufkova, Matej Bezdicek, Marketa Nykrynova, Iva Kocmanova, Petra Kubackova, Jana Hanslikova, Karolina Fejkova, Jiri Mayer, Martina Lengerova

**Affiliations:** a Department of Internal Medicine - Haematology and Oncology, University Hospital Brno, Brno, Czech Republic; b Department of Internal Medicine - Haematology and Oncology, Faculty of Medicine, Masaryk University, Brno, Czech Republic; c Department of Biomedical Engineering, Brno University of Technology, Brno, Czech Republic; d Department of Clinical Microbiology and Immunology, University Hospital Brno, Brno, Czech Republic; Veterans Affairs Northeast Ohio Healthcare System

**Keywords:** *Pseudomonas aeruginosa*, high-resolution melting, mini-MLST, molecular epidemiology, strain typing

## Abstract

The Pseudomonas aeruginosa population has a nonclonal epidemic structure. It is generally composed of a limited number of widespread clones selected from a background of many rare and unrelated genotypes recombining at high frequency. Due to the increasing prevalence of nosocomial infections caused by multidrug-resistant/extensively drug-resistant (MDR/XDR) strains, it is advisable to implement infection control measures. Pulsed-field gel electrophoresis (PFGE) and multilocus sequence typing (MLST) are considered the gold standard methods in bacterial typing, despite being limited by cost, staff, and instrumental demands. Here, we present a novel mini-MLST scheme for P. aeruginosa rapid genotyping based on high-resolution melting analysis. Using the proposed mini-MLST scheme, 3,955 existing sequence types (STs) were converted into 701 melting types (MelTs), resulting in a discriminatory power of *D* = 0.993 (95% confidence interval [CI], 0.992 to 0.994). Whole-genome sequencing of 18 clinical isolates was performed to support the newly designed mini-MLST scheme. The clonal analysis of STs belonging to MelTs associated with international high-risk clones (HRCs) performed by goeBURST software revealed that a high proportion of the included STs are highly related to HRCs and have also been witnessed as responsible for serious infections. Therefore, mini-MLST provides a clear warning for the potential spread of P. aeruginosa clones recognized as MDR/XDR strains with possible serious outcomes.

**IMPORTANCE** In this study, we designed a novel mini-MLST typing scheme for Pseudomonas aeruginosa. Its great discriminatory power, together with ease of performance and short processing time, makes this approach attractive for prospective typing of large isolate sets. Integrating the novel P. aeruginosa molecular typing scheme enables the development and spread of MDR/XDR high-risk clones to be investigated.

## INTRODUCTION

Pseudomonas aeruginosa is a pathogen with high clinical relevance, including life-threatening infections in immunocompromised patients and patients with cystic fibrosis. It is also a common cause of health care-associated infections (HCAIs), such as ventilator-associated pneumonia, urinary tract infections, and surgical site infections (1). Its large and plastic genome comprises multiple virulence and resistance determinants and also facilitates its remarkable adaptability to unfavorable environments, including hospital environments with possible consequent transmission (2, 3). In addition, cross-transmission from patient to patient and endogenous sources have been regarded as relevant routes of P. aeruginosa acquisition as well (4). Pseudomonadal infections are difficult to treat because the pathogen is intrinsically resistant to many antibiotics due to low outer membrane permeability and readily acquired multidrug resistance that further complicates treatment (5). A significant proportion of multidrug resistance or even extensive drug resistance (MDR/XDR) in P. aeruginosa strains have been witnessed in increasing prevalence in the last few decades (6). However, in the period of 2016 and 2020, European Union/European Economic Area surveillance showed decreasing trends in P. aeruginosa antimicrobial resistance, although with major geographical differences (7). Still, at the global level, carbapenem-resistant P. aeruginosa has been listed as a pathogen of critical priority requiring research and development of new antibiotics (8).

Genotyping represents an important process that enables clinically important bacterial strain characterization and surveillance. Understanding pathogen relatedness is essential for studying HCAI epidemiology and designing rational pathogen control methods. For P. aeruginosa genotyping, various molecular methods have been used (9). These include pulsed-field gel electrophoresis of SpeI-restricted genomic DNA (PFGE-SpeI) (10), multiple-locus variable number of tandem repeat analysis (MLVA) (11), and multilocus sequence typing (MLST) (12), followed by the technologically advanced whole-genome sequencing (WGS)-based methods, such as single nucleotide variant (SNV) analysis and core genome MLST (cg-MLST) and accessory genome MLST (ag-MLST) (13). Among those, possibly the most popular approach for studying P. aeruginosa populations is MLST.

For proper genotyping interpretation, the evolution dynamics typical for each bacterial species is important to consider. As for P. aeruginosa, the population structure is referred to as a nonclonal epidemic structure composed of a large quantity of rare strains and a limited number of highly successful epidemic clones (14, 15). It has been stated that MDR and especially XDR strains have a low clonal diversity and belong to so-called high-risk clones (HRCs), contrary to susceptible isolates that are mostly represented by numerous different genotypes (16, 17). Despite the highest level of standardization, MLST is demanding on both time and staff and high in cost. Methods including PCR followed by high-resolution melting (HRM) instead of sequencing have become popular in epidemiology (18) because they are easily accessible for routine use. HRM has been used to modify MLST, known as “mini-MLST” or “minim typing.” Mini-MLST targets the same genes as MLST, but sequencing is replaced by HRM. The acquired divergent melting curves represent individual melting alleles, and a combination of individual loci’s alleles enables the isolate to be assigned as a melting type (MelT). So far, mini-MLST schemes have been published for Staphylococcus aureus (19), Klebsiella pneumoniae (20), Streptococcus pyogenes (21), Enterococcus faecium (22), Campylobacter jejuni (23), and Escherichia coli (24). Here, we describe a novel mini-MLST scheme for P. aeruginosa as an alternative method for rapid genotyping suitable for routine clinical practice.

## RESULTS

### Method design.

The candidate regions for mini-MLST design identified by EasyPrimer were subjected to *in silico* analyses. We assumed an optimal amplicon length of 70 to 200 bp. For *aroE* locus, no appropriate conserved sites available for primer design were detected; thus, this region was excluded from future analyses. Simpson’s Index of Diversity (*D*) was calculated for each predesigned locus, and those with the highest discriminatory ability were chosen for the following laboratory experiments. Finally, a set of six loci containing highly informative single nucleotide polymorphisms (SNPs) meeting our criteria were found in six different MLST loci (Table 1). The *D* values calculated for the designed mini-MLST loci calculated against MLST sequence variability were (locus name plus first and last position of mini-MLST locus within MLST sequence): *acsA325-393* (*n* = 274; *m* = 9; *D* = 0.764; 95% confidence interval [CI], 0.732 to 0.795), *guaA88-225* (*n* = 219; *m* = 10; *D* = 0.734; 95% CI, 0.705 to 0.763), *mutL18-82* (*n* = 305; *m* = 8; *D* = 0.688; 95% CI, 0.661 to 0.715), *nuoD23-95* (*n* = 153; *m* = 6; *D* = 0.656; 95% CI, 0.596 to 0.716), *ppsA85-136* (*n* = 209; *m* = 6; *D* = 0.679; 95% CI, 0.646 to 0.712), and *trpE270-378* (*n* = 315; *m* = 7; *D* = 0.757; 95% CI, 0.738 to 0.776), where *n* stands for the total number of MLST alleles, and *m* stands for the total number of predicted mini-MLST alleles.

**TABLE 1:** Details of the six loci used in P. aeruginosa mini-MLST typing scheme^*a*^

Name	Primer sequence (5′ to 3′)	Amplicon length (bp)	Predicted mini-MLST allele (no. of associated STs)^*b*^
*acsA*	CCTGCCTGATGACCCCG	107	47 (1); 48 (5); 49 (42); 50 (34); 51 (107); 52 (56); 53 (18); 54 (10); 55 (1)
	GTGGACAACCTCGGCAACCT ^*c*^		
*guaA*	CCAACTGACCTGCGTGTTC	177	73 (1); 84 (1); 85 (1); 88 (3); 90 (1); 91 (11); 92 (41); 93 (75); 94 (73); 95 (12)
	GAGAAGCGCAAGATCATCGG		
*mutL*	AGACCGAGTTCGACCAT ^*c*^	96	28 (1); 36 (8); 37 (1); 38 (13); 39 (113); 40 (48); 41 (118); 42 (3)
	ATGGTCTTGCCGTTGTG		
*nuoD*	TTCCTCAACCTCGGCCCGA	110	46 (2); 47 (13); 48 (30); 49 (80); 50 (26); 51 (2)
	GGAGATCGGCTACCACCA		
*ppsA*	CTGCTGAAAGAGAAGGGGAC	92	30 (1); 36 (22); 37 (78); 38 (84); 39 (1); 40 (23)
	AAGGTGATCAACGACGTGTC		
*trpE*	ACTCCAACGTCATGCACATC	149	75 (4); 76 (26); 77 (80); 78 (86); 79 (98); 80 (7); 81 (14)
	AGATCATCGACGAGCTGGAG		

a MLST, multilocus sequence typing; ST, sequence type.

b By July 13, 2022, *n* = 3,955 STs.

c Primer for MLST used (12).

### Mini-MLST scheme completion.

A conversion key was generated to enable mini-MLST allele numbers to be combined with the final MelT and also to convert between sequence types (STs) and MelTs. In July 2022, 3,955 existing STs were converted into 701 MelTs. The *D* expressing the total mini-MLST discriminatory power against the MLST database yielded *D* = 0.993 (95% CI, 0.992 to 0.994).

### Method validation and typing of collected isolate set.

**FIG 1:**
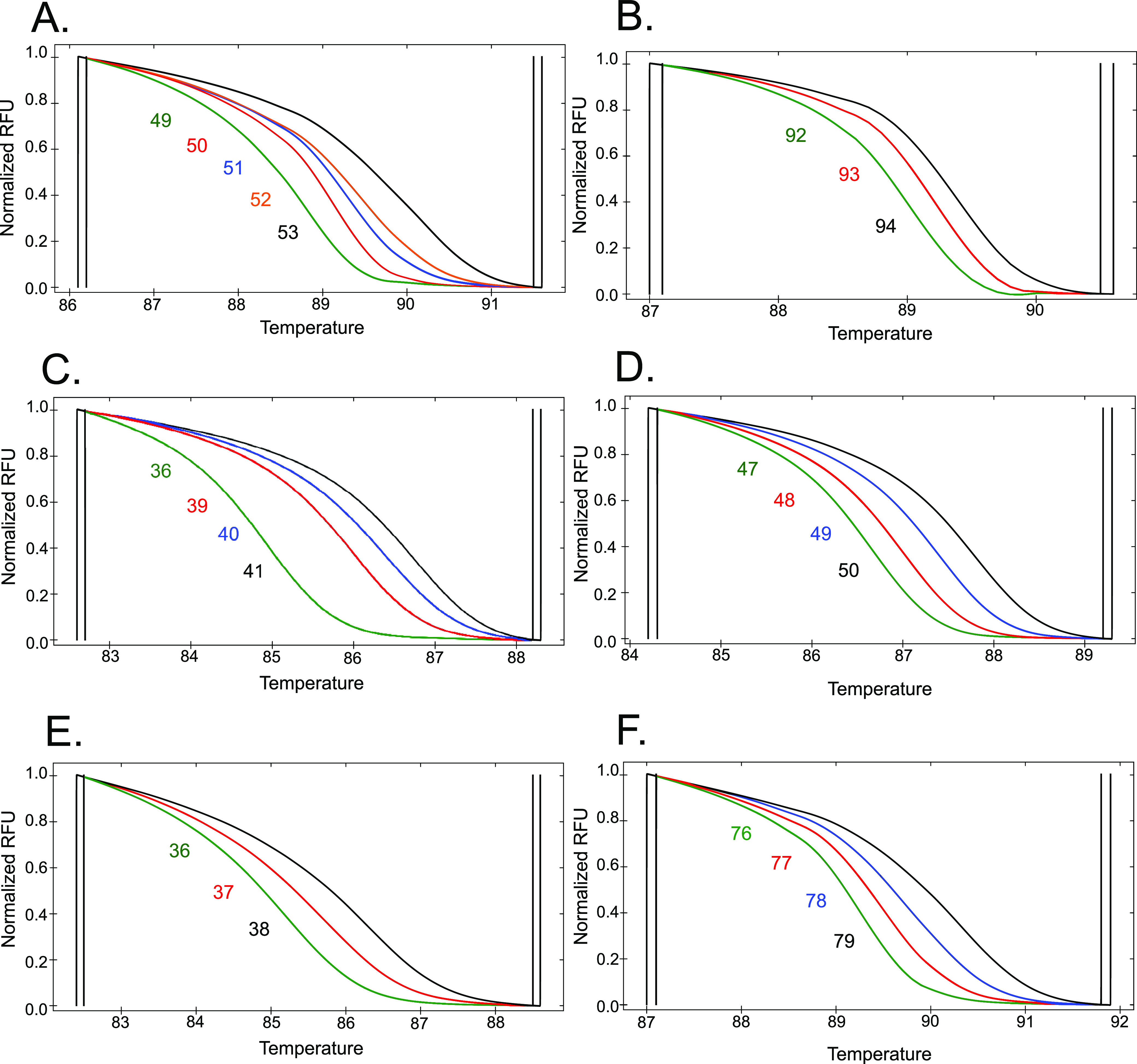
Normalized melting curves for six mini-multilocus sequence typing (MLST) loci detected during method validation. The allele numbers are assigned to the associated melting curves according to the G + C content. (A) Locus *acsA*, 5 of 9 predicted alleles (alleles 49 to 53). (B) Locus *guaA*, 3 of 10 predicted alleles (alleles 92, 93, and 94). (C) Locus *mutL*, 4 of 8 predicted alleles (alleles 36 and 39 to 41). (D) Locus *nuoD*, 4 of 6 predicted alleles (alleles 47 to 50). (E) Locus *ppsA*, 3 of 6 predicted alleles (alleles 36 to 38). (F) Locus *trpE*, 4 of 7 predicted alleles (alleles 76, 77, 78, and 79). RFU show the relative fluorescence unit values.

The HRM curves for each six mini-MLST loci were obtained for 191 of 200 P. aeruginosa isolates. Two isolates were repeatedly not amplifying properly, and the HRM curves from seven isolates had a nonstandard shape and differed from the remaining HRM curves in at least one mini-MLST locus. These nine isolates were excluded from further analyses. The set of positive controls we observed during scheme design is shown in Fig. 1. Of 191 P. aeruginosa isolates, 74 different MelTs were determined as summarized in Table 2.

**TABLE 2:** Summary of mini-MLST typing of 191 P. aeruginosa isolates^*a*^

MelT	No. of isolates	Proportion (%)	MelT associated with HRC	Used for WGS (no. of used isolates)
MelT127	11	5.8	ST244	Yes (4)
MelT306	11	5.8	No	Yes (3)
MelT384	11	5.8	No	Yes (2)
MelT375	10	5.2	No	Yes (3)
MelT491	9	4.7	ST308	Yes (3)
MelT417	7	3.7	No	No
MelT247	6	3.1	ST235	No
MelT355	6	3.1	No	No
MelT524	6	3.1	No	No
MelT76	4	2.1	ST111/ST654	Yes (3)
MelT78	4	2.1	ST175	No
MelT251	4	2.1	No	No
MelT308	4	2.1	No	No
MelT418	4	2.1	No	No
MelT425	4	2.1	No	No
Others^*b*^	59	47.1	NA^*c*^	No

a Table shows a summary of mini-MLST typing of 191 P. aeruginosa isolates obtained at University Hospital Brno, Czech Republic, between March 2020 and October 2021. HRC, high-risk clone; MelT, melting type; NA, not available; WGS, whole-genome sequencing.

b Includes MelTs with less than 2% of isolates.

c Among less abundant MelTs, MelTs associated with HRCs were present: MelT602 associated with ST233 (*n* = 1), MelT618 associated with ST357 (*n* = 2), and MelT340 associated with ST298 (*n* = 3). MelT52 associated with ST277 was not present in our set of isolates.

### Whole-genome sequencing.

**FIG 2:**
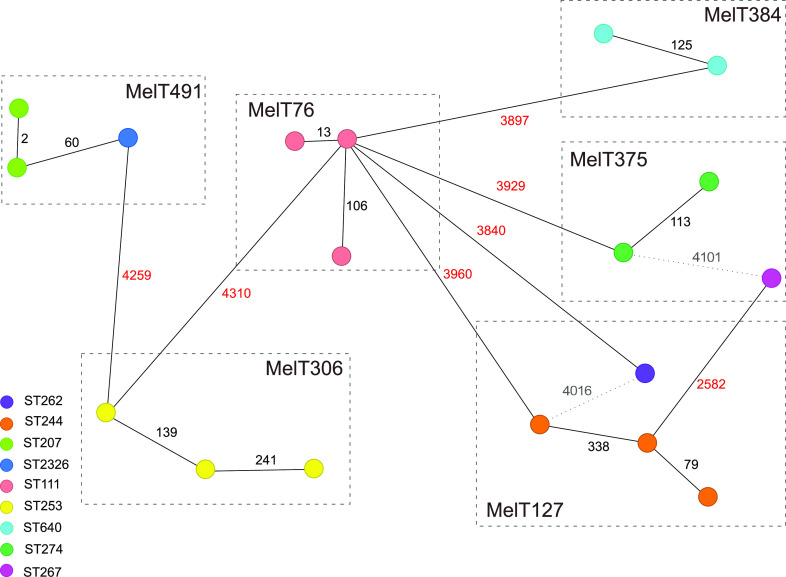
Minimum spanning tree showing core genome MLST (cg-MLST) and accessory genome MLST (ag-MLST) of 18 P. aeruginosa isolates. Each isolate is represented by a unique node. Lines between circles show the number of allelic differences with those representing distance among different melting types (MelTs) highlighted in red. Sequence types (STs) are color coded, and MelTs are indicated by frame. The gray dotted line numbers show the distance among distant STs clustered into one MelT.

Mini-MLST’s discriminatory ability was tested using WGS on a subset of 18 P. aeruginosa isolates belonging to the dominating MelTs and MelTs that refer to international HRCs. For WGS analysis, we used allelic difference comparison with cg- and ag-MLST since it takes homologous recombination into consideration, contrary to SNV analysis that counts every single nucleotide change as an additional evolutionary event. Fig. 2 shows a minimum spanning tree (MST) based on 18 isolates’ allelic profiles. The distances between different MelTs were higher than 2,000 allele differences. Within one MelT, closely related STs were present (ST2326 and ST207 in MelT491 differ in one MLST allele). On the other hand, we observed distant STs grouped together (ST262 and ST244 in MelT127, and ST267 and ST274 in MelT375).

### HRC investigation.

**FIG 3:**
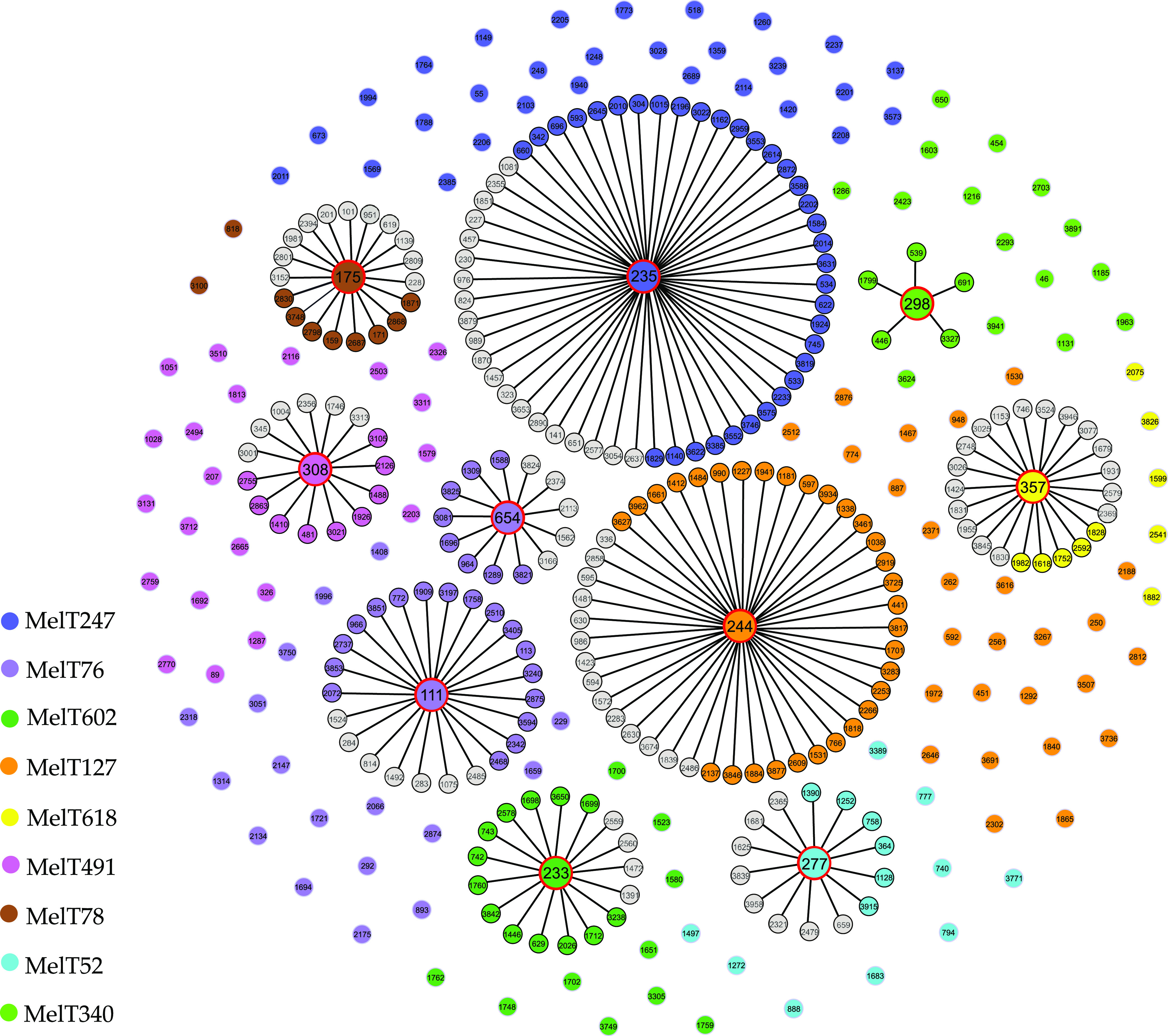
Genetic relationships among different STs that belong to MelTs associated with international high-risk clones (HRCs) (produced by goeBURST algorithm). Each circle corresponds to an individual ST. HRC STs as defined by del Barrio-Tofiño et al. (17) are represented by large circles with a red halo. Single-loci variant STs are connected with its founder ST with a solid line. Colors are assigned according to the MelT. Gray indicates that the ST is assigned to an MelT other than the founder.

The BURST analysis of all STs revealed significant heterogeneity among STs characteristic for P. aeruginosa population structure (Fig. S1). First, clusters containing international HRCs as cluster founders and related single-loci variant STs were inspected. Within those clusters, MelTs corresponding to founder HRC STs were color coded (Fig. 3). Single-loci variant STs affiliated with other MelTs than the founder remained color free. The proportion of single-loci variant STs belonging to the same MelT as the cluster founder was determined (Table 3). Second, it was determined whether MelTs associated with HRCs contained nonrelated STs. Those were depicted as singletons with no connection lines to cluster founders.

**TABLE 3:** Overview of international HRCs examined by BURST analysis

HRC	Associated MelT	No. of STs in HRC cluster^*a*^	No. (proportion) of STs in HRC cluster belonging to HRC MelT^*b*^	No. of STs in MelT	No. (proportion) of singletons belonging to MelT
ST235	MelT247	55	35 (63.6%)	64	29 (45.3%)
ST111	MelT76^*c*^	25	18 (72.0%)	44	17 (38.6%)
ST233	MelT602	18	14 (77.8%)	24	10 (41.7%)
ST244	MelT127	45	31 (68.9%)	57	26 (45.6%)
ST357	MelT618	23	6 (26.1%)	11	5 (45.5%)
ST308	MelT491	16	10 (62.5%)	31	21 (67.7%)
ST175	MelT78	20	9 (45.0%)	11	2 (18.2%)
ST277	MelT52	15	7 (46.7%)	16	9 (56.3%)
ST654	MelT76^*c*^	14	9 (64.3%)	44	17 (38.6%)
ST298	MelT340	6	6 (100.0%)	21	15 (71.4%)

a The HRC represents the founder of the cluster. All STs that differ in one allele are included in the cluster.

b Including founder ST.

c ST111 and ST654 belong to the same MelT: MelT76.

## DISCUSSION

Despite MLST currently being considered an effective method to study bacteria’s molecular epidemiology and population structure, it is actually far from routinely used in local hospital laboratories, mainly due to the time and cost demands. Contrary to sequencing-based methods, incorporating HRM analysis promises an inexpensive, fast, and robust alternative approach (18).

In this study, we have proposed a novel mini-MLST scheme for prospective P. aeruginosa molecular typing. It includes six multi-SNPs targeting PCR followed by HRM analysis, resulting in a great discriminatory ability. Among the major advantages of this typing approach are its cost-effectiveness, rapid performance, robustness, and good reproducibility. Moreover, we optimized the reaction mixture and run parameters to existing K. pneumoniae and E. coli mini-MLST schemes that enable simultaneous typing for these clinically important bacterial species and a significant decrease in the laboratory work and analysis time demands. The design based on MLST loci enables results comparable with MLST data to be generated through the translation MelT key.

To validate mini-MLST for routine clinical practice, we tested the novel scheme on 191 clinical isolates of different sites of origin collected at University Hospital Brno, Brno, Czech Republic. The typing revealed a heterogenous population containing 74 MelTs supporting the consensus of nonclonal epidemic population structure (15). Isolate WGS with subsequent cg- and ag-MLST proved a long allelic distance among different MelTs, as well as a shorter distance between isolates of the same MelTs and STs (Fig. 2). Within MelT491, closely related STs were present: single loci variants ST2326 and ST207 with a WGS allelic distance of 60. On the other hand, we observed distant STs grouped together by mini-MLST. MelT127 cluster isolates belonging to ST262 and ST244. Those STs are triple-loci variants; however, their WGS allelic distance reaches 4,016. MelT375 contains ST267 and ST274 isolates; these differ in six MLST genes, and their WGS allelic distance is 4,101. This disparity is caused by the fact that different MLST alleles can share the same G + C content and produce the same HRM curve despite having a different sequence.

Incorporating mini-MLST to prospective P. aeruginosa hospital monitoring allows strains that arise from rare heterogenous population to be focused on. The successful clones associated with defined MDR/XDR profiles have been described and denominated international HRCs. In our study, we tested whether the identification of the MelTs associated with HRCs suggests a high possibility of HRC presence or whether the presence of STs closely related to them. BURST analysis showed the ST relationships among clusters that include HRCs (Fig. 3). In those clusters, single-loci variant STs belonging to the same MelT as cluster founder ST were appointed. Also, single-loci variant STs classified into different further nonspecified MelTs due to different melting profile were included. The proportion of single-loci variant STs belonging to a founder MelT compared to the all single-loci variant STs, including those assigned to other MelTs, was determined (Table 3). We inspected all STs clustered by mini-MLST to the same MelTs; however, they are not parts of HRC clusters (they differ in 2 MLST loci or more). The results differed significantly between different HRC clusters. ST235 is the most relevant HRC that has been identified as a highly successful MDR clone worldwide. There are many diverse single-loci variant STs that have independently arisen from ST235 (25); 63.6% of them belong to MelT247. However, identifying this MelT indicates CC235 in only 54.7%; in 45.3%, it indicates singleton STs that are not related to ST235. In CC298, 100% of single-loci variant STs belong to associated MelT340. However, this MelT gathers STs unrelated to ST298 in 71.4%, thus determining that an isolate belonging to this MelT is not highly indicative to the presence of HRC. On the other hand, identifying MelT78 is indicative of CC175 in 81.8%. Only two STs belonging to this MelT are not related to ST175. Within CC175, STs of MelT78 represent 45.0% in proportion to other nonspecified MelTs. The BURST analysis summary (Table 3) may serve to advise whether or not the detected MelT warns us about the possible occurrence of dangerous of P. aeruginosa clones.

To conclude, mini-MLST represents an effective method for P. aeruginosa molecular typing. Its good reproducibility and easy performance allow prospective typing for large sets of isolates while reaching a great discriminatory power. Integrating a novel P. aeruginosa mini-MLST scheme enables monitoring and limiting the occurrence and spread of newly arisen clones within health care facilities. The detection of MelTs associated with HRCs is also indicative of the presence of MDR/XDR HRCs.

## MATERIALS AND METHODS

### Method design: mini-MLST loci identification and primer design.

The P. aeruginosa MLST scheme for 3,955 STs (by July 13, 2022) including *acsA*, *aroE*, *guaA*, *mutL*, *nuoD*, *ppsA*, and *trpE* allele sequences was obtained from the PubMLST.org website using BIGSdb software (26). All alleles within each gene were aligned using MEGA7 software (27). The informative MLST gene regions with high sequence variability were identified using EasyPrimer tool (28), taking into consideration A⇆T and C⇆G nucleotide changes that cannot be detected by HRM analysis. Subsequently, the primers were designed to flank the selected informative regions using Primer3 (29).

### Mini-MLST scheme completion: establishing MelT key.

An in-house MELT2MELT algorithm based on MATLAB software was used to generate a MelT key as described by Bezdicek et al. (24). The final MelT key is available at www.cmbgt.cz/mini-mlst/t6353 and is regularly updated as new STs are consistently released.

### Statistical analysis.

To measure the discriminatory ability of the novel mini-MLST scheme, Simpson’s index of diversity (*D*) was defined (30) using the Comparing Partitions website (www.comparingpartitions.info).

### Isolate collection and DNA isolation.

In total, 200 P. aeruginosa clinical isolates were subjected to the newly designed mini-MLST method. All of the isolates were collected throughout the University Hospital Brno, Brno, Czech Republic, between March 2020 and October 2021 and were identified using matrix-assisted laser desorption/ionization time-of-flight mass spectrometry (MALDI-TOF MS). The pure culture colonies were suspended in sterile water and stored at −20°C. The genomic DNA was isolated using Chelex 100 resin (Bio-Rad, USA). The bacterial cultures were homogenized in 100 μL of 5% wt/vol Chelex 100 resin with vortex. The suspensions were incubated for 10 min in 99°C and centrifuged for 2 min at 15,500 relative centrifugal force. The supernatants containing genomic DNA were transferred into clean microtubes and stored at −20°C for further analyses.

### Method validation: real-time PCR and HRM.

The mini-MLST laboratory protocol was designed to be applicable for K. pneumoniae (31), E. coli (24), and P. aeruginosa typing simultaneously in the same PCR run. Real-time PCRs contained 10 μL 2× SensiFAST HRM mix (Bioline, UK), 0.4 μM each primer, 1 μL of DNA (30 ng/μL), and deionized water to a final volume of 20 μL. The thermal cycling parameters were 95°C for 3 min; 40 cycles of 95°C for 5 s, 65°C for 10 s, and 72°C for 20 s; 95°C for 2 min; and 50°C for20 s, followed by HRM ramping from 70 to 95°C, increasing by 0.2°C at each step. For the analyses, a Bio-Rad CFX96 real-time PCR detection system (Bio-Rad, USA) was used. Samples with unique HRM curves were subjected to Sanger sequencing using MLST primers to obtain G + C content in the mini-MLST region. For *acsA*, *guaA*, *mutL*, *ppsA*, and *trpE* genes, MLST was performed as previously described by Curran et al. (12). For *aroE* and *nuoD* genes, protocol by van Mansfeld et al. (32) was used. PCR products were purified using ExoSAP-IT (Thermo Fisher Scientific, USA) and sequenced using BigDye Terminator version 1.1 (Thermo Fisher Scientific, USA) with an ABI3130 genetic analyzer (Thermo Fisher Scientific, USA) and sequencing analysis version 5.4 software. In total, 32 isolates were subjected to complete conventional MLST analysis and used as positive controls during mini-MLST. Melting curves of unknown samples were allocated to the positive controls with the same melting profile, and the allele number was assigned according to the G + C content.

### Whole-genome sequencing.

WGS was used to support the mini-MLST discriminatory ability. The WGS library was prepared using KAPA HyperPrep kits (Roche, Switzerland). The Illumina MiSeq platform was used for WGS, and 250-bp paired-end sequencing was performed. The obtained reads were quality checked using FastQC version 0.11.5 (Babraham Bioinformatics, UK) and assembled using Burrows-Wheeler Aligner version 0.7.17 (33). Ridom SeqSphere+ (Ridom, Germany) P. aeruginosa seed genome strain PAO1 (NC_002516.2) was used as the reference genome. To remove unmapped reads, reads with poor quality, and duplicates, SAMtools version 1.9 was used (34). After reference mapping, all positions with less than 10% coverage, and all ambiguous positions (less common bases represented at least 10% of bases in the target position) were removed from further analyses. SAMtools version 1.9 was used together with BCFtolls version 1.9 software to obtain consensus sequences (35). With SeqSphere+ version 7.8 (Ridom, Germany), the genomes were compared with a gene-by-gene approach using an incorporated P. aeruginosa cg-MLST scheme and P. aeruginosa ag-MLST scheme comprising 3,867 cg- and 1,647 ag-genes, respectively (13). A MST was constructed to visualize the allelic differences between the isolates.

### Clonal analysis.

All STs profiles (3,955 STs, by July 13, 2022) were obtained from the PubMLST website (https://pubmlst.org/organisms/pseudomonas-aeruginosa). To identify groups of related STs, we compared the STs by the global optimal based upon the related ST algorithm (goeBURST) (36) at the level of single-loci variants to create clonal clusters (CCs). To provide a graphical representation of the evolutionary relationships between them, we used Phyloviz 2.0 software (37). The top 10 international HRCs as stated by del Barrio-Tofiño et al. (17) were identified, and their CCs were obtained. Only STs with one allele difference to the founder HRC were included in each CC.

### Data availability.

The raw sequencing data were deposited in the SRA database under the BioProject accession number PRJNA855568.
