# Supplementary figures and images for: Rapid Identification of Pseudomonas aeruginosa International High-Risk Clones Based on High-Resolution Melting Analysis
Source: Microbiol Spectr. 2023 Jan 11;11(1):e03571-22. doi: 10.1128/spectrum.03571-22 (PMC9927482; doi:10.1128/spectrum.03571-22)

Fig. S1. Clustering analysis by the goeBURST algorithm performed for 3,955 *P. aeruginosa* STs

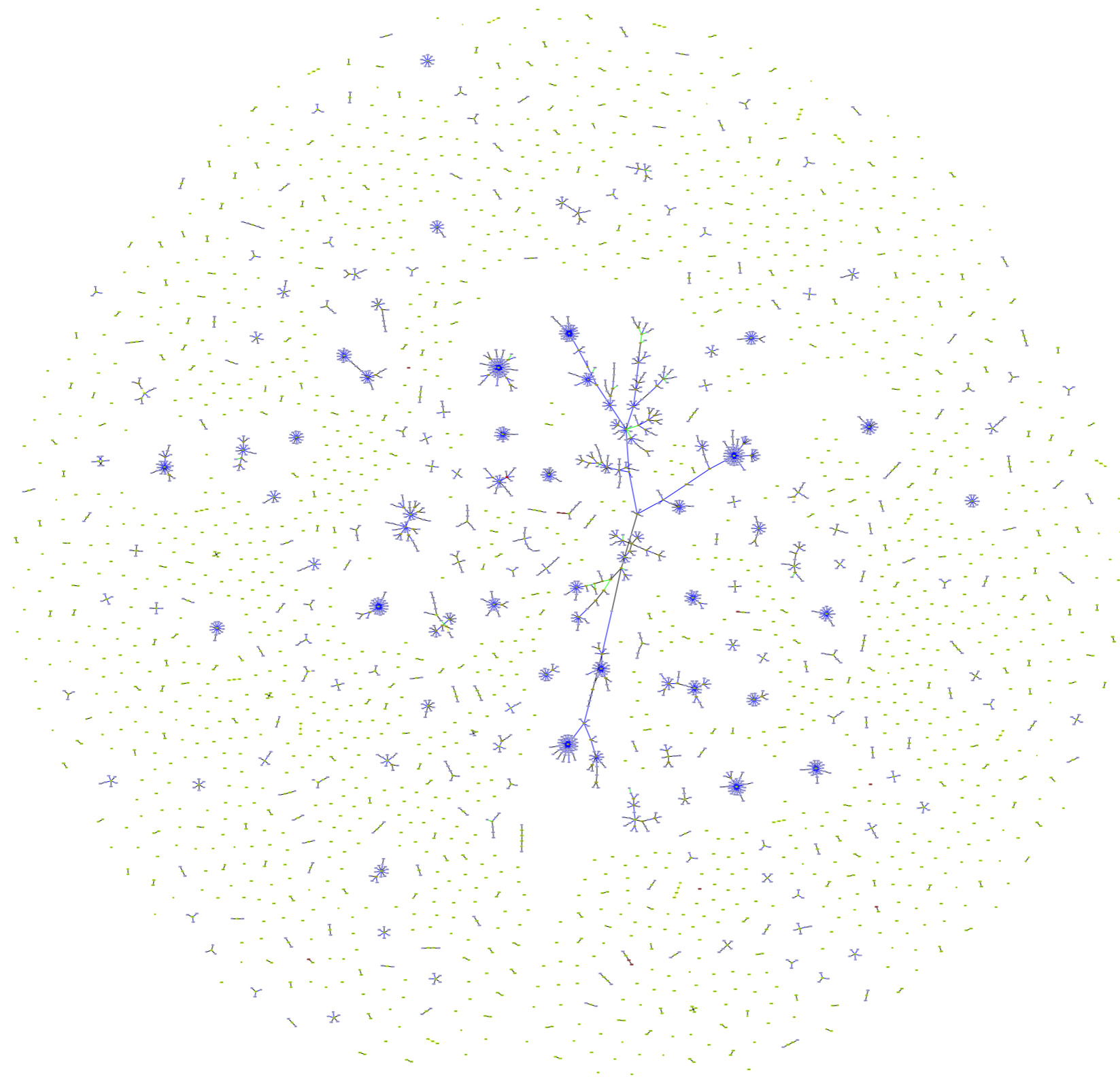

Supplement: Supplemental file 1 — Fig. S1. Download spectrum.03571-22-s0001.pdf, PDF file, 5.2 MB [file spectrum.03571-22-s0001.pdf]
